# Can Older Adults Accurately Perceive Their Hand Function?

**DOI:** 10.1093/geroni/igaf122.2865

**Published:** 2025-12-31

**Authors:** Jayne Parry, Amanda Mechaber, Rachel Logue Cook, Susan Brown

**Affiliations:** University of Michigan, Ann Arbor, Michigan, United States; University of Michigan, Ann Arbor, Michigan, United States; University of Michigan, Ann Arbor, Michigan, United States; University of Michigan, Ann Arbor, Michigan, United States

## Abstract

Older adults have been shown to overestimate their ability to control balance and locomotion (Kawasaki & Tozawa, 2020; Martin et al., 2021). Despite the importance of upper limb ability for daily living, it is unknown whether older adults also overestimate manual ability. The purpose of this study was to determine whether older adults overestimate hand function compared to objective hand measures. Perception and physical measures of hand function were assessed in 28 community-dwelling older adults (mean age 75.2 ± 3.3 y/o). An adapted survey (Logue Cook et al, 2024) assessed participants’ perceptions of hand function. Strength and dexterity of both hands were assessed using standard dynamometry, the Purdue Pegboard Test, and the Jebsen Taylor Hand Function Test. Two thirds of participants (66.7%) reported their current hand function to be better than the average for their age while one third (33.3%) thought their function was average. None of the participants reported their hand function as worse than average. However, 40.7% of individuals had at least one objective hand measure that fell below one standard deviation of the group mean. Of this group, 55% reported their hand function to be better than average. Objective measures showed age-related declines in hand function despite older adults perceiving their hand function as average or better than average. A tendency to overestimate hand function using self-reported surveys underscores the importance of including more objective measures when monitoring age-related changes in hand health.

